# Endoscopic tattooing of early colon carcinoma enhances detection of lymph nodes most prone to harbor tumor burden

**DOI:** 10.1007/s00464-016-5026-3

**Published:** 2016-06-20

**Authors:** Iban Aldecoa, Carla Montironi, Nuria Planell, Maria Pellise, Gloria Fernandez-Esparrach, Angels Gines, Salvadora Delgado, Dulce Momblan, Leticia Moreira, Maria Lopez-Ceron, Natalia Rakislova, Graciela Martinez-Palli, Jaume Balust, Josep Antoni Bombi, Antonio de Lacy, Antoni Castells, Francesc Balaguer, Miriam Cuatrecasas

**Affiliations:** 1 Pathology Department-Centre de Diagnostic Biomedic (CDB), Hospital Clínic, University of Barcelona (CIBERehd) and Banc de Tumors-Biobanc Clinic-IDIBAPS-XBTC, Escala 3, Planta 5, Villarroel 170, 08036 Barcelona, Spain; 2 Bioinformatics Unit, CIBERehd, Barcelona, Spain; 3 Gastroenterology Department, Hospital Clinic, IDIBAPS, Centro de Investigación Biomédica en Red de Enfermedades Hepáticas y Digestivas (CIBERehd), University of Barcelona, Barcelona, Catalonia Spain; 4 Surgery Department, Hospital Clinic, Barcelona, Spain; 5 Anestesiology Department, ICMDiM, Hospital Clinic-IDIPAPS, Barcelona, Spain

**Keywords:** Colorectal cancer, Lymph nodes, Endoscopic tattooing, India ink, OSNA

## Abstract

**Background:**

Colorectal cancer (CRC) screening programs result in the detection of early-stage asymptomatic carcinomas suitable to be surgically cured. Lymph nodes (LN) from early CRC are usually small and may be difficult to collect. Still, at least 12 LNs should be analyzed from colectomies, to ensure a reliable pN0 stage. Presurgical endoscopic tattooing improves LN procurement. In addition, molecular detection of occult LN tumor burden in histologically pN0 CRC patients is associated with a decreased survival rate. We aimed to study the impact of presurgical endoscopic tattooing on the molecular detection of LN tumor burden in early colon neoplasms.

**Methods:**

A prospective cohort study from a CRC screening-based population was performed at a tertiary academic hospital. LNs from colectomies with and without preoperative endoscopic tattooing were assessed by two methods, hematoxylin and eosin (HE), and RT-LAMP, to detect tumor cytokeratin 19 (CK19) mRNA. We compared the amount of tumor burden and LN yields from tattooed and non-tattooed specimens.

**Results:**

HE and RT-LAMP analyses of 936 LNs were performed from 71 colectomies containing early carcinomas and endoscopically unresectable adenomas (8 pT0, 17 pTis, 27 pT1, 19 pT2); 47 out of 71 (66.2 %) were tattooed. Molecular positivity correlated with the presence of tattoo in LN [*p* < 0.001; OR 3.1 (95 % CI 1.7–5.5)]. A significantly higher number of LNs were obtained in tattooed specimens (median 17 LN vs. 14.5 LN; *p* = 0.019).

**Conclusions:**

Endoscopic tattooing enables the analysis of those LNs most prone to harbor tumor cells and improves the number of LN harvested.

**Electronic supplementary material:**

The online version of this article (doi:10.1007/s00464-016-5026-3) contains supplementary material, which is available to authorized users.

Endoscopic tattooing was originally introduced as a reliable and accurate method to localize colonic lesions on follow-up colonoscopies, i.e., incompletely resected polyps or scars from prior polypectomies [1]. Its use has been extended with the introduction of laparoscopic and robotic surgery to locate lesions and determine the extent of colonic resection [1–4]. In addition, colorectal cancer (CRC) population screening programs have increased the detection of early-stage asymptomatic CRC and malignant polyps [5]. This has led to new challenging diagnostic and management issues, including the need to adequately define the underlying malignant potential of a certain polyp [6], the decision of endoscopic control or surgical treatment, and the indication for postoperative adjuvant chemotherapy.

Surgically removed early colorectal tumors face the challenge of assessing the lymph node (LN) metastatic potential of a single case, based on the information included in the histopathology report [7]. Considering that LN staging is an accurate prognostic factor, there is a broad consensus on the need to achieve the highest number of LNs [7–9]. In order to increase histological diagnostic sensitivity, gross LN enhancement with nodal revealing solutions [10] and molecular LN staging assessment have been proposed [11–18]. Nevertheless, despite the evidences of impaired survival rates associated with the molecular detection of tumor cells in regional LN of stage I–II CRC patients [12, 14, 19], LN molecular analysis is not performed on a daily basis, mainly due to its high costs and time-consuming nature.

Moreover, the impact of presurgical submucosal tattooing on the LN yield obtained is controversial [9, 20–22]. Methodological differences in the type and number of LN studied, as well as the different aims of the studies, may account for the discrepancies, i.e., some authors have used India ink to identify sentinel lymph nodes (SLN), advocating that it may help to identify metastatic LN, while others have used it for LN mapping and increasing the number of LN [20, 23–25].

The aim of this study was to assess the impact of endoscopic tattooing on the molecular detection of LN tumor burden in early colon neoplasia. We used the molecular assay one-step nucleic acid amplification (OSNA, Sysmex Corporation, Kobe, Japan), which is based on the quantitative detection of cytokeratin 19 (CK19) mRNA. This technique is widely used in the evaluation of breast cancer SLN [26] and is being introduced to assess regional LN in colon [27, 28], lung [29], endometrial [30], and thyroid cancer [31]. In addition, we also aimed to assess the enhancement of LN procurement in tattooed colectomies.

## Materials and methods

### Patients

From May 2012 to December 2013, we included all patients submitted to surgery for colon carcinomas or endoscopically unresectable adenomas diagnosed at our institution. The study was performed in the context of a population-based CRC screening program using fecal immunochemical test (FIT) (OC-Sensor^®^, Eiken, Japan; cutoff, ≥20 μg of hemoglobin/g of feces). FIT was offered every 2 years to asymptomatic individuals between 50 and 69 years old.

This study is focused on the molecular identification of LN metastasis in early colon neoplasms (pT0–2) and the influence of tattooing in this setting. We included individuals over 18 years old with endoscopically unresectable adenomas, malignant polyps with confirmed pT1 carcinoma containing adverse prognostic factors associated with LN metastasis (i.e., the presence of at least one of the following features: poor differentiation, lymphovascular invasion, high-grade tumor budding, tumor margin ≤1 mm, and submucosal invasion ≥2 mm), and pT1–2 stage colon carcinomas. pT3–4 carcinomas were excluded since they are seldom tattooed at our institution as they are regarded as large infiltrating masses easy to be surgically localized. Other exclusion criteria were tumor infiltration of the mesocolon fat on gross examination, rectal tumors, synchronous colorectal carcinomas, appendicular carcinomas, the presence of colonic stent, inflammatory bowel disease or other malignancies, and reception of surgical specimen immersed in formalin.

### Ethical considerations

The study was approved by the Ethics Committee of Hospital Clinic of Barcelona, Spain. All patients signed and kept a copy of the informed consent document for participation in the study. Another copy was kept with the patient’s clinical files.

### Study procedures

#### Colonoscopy and tattooing

Colonoscopies were performed on patients with a positive FIT result. Our institution has a high-quality endoscopy unit dedicated to screening and follow-up colonoscopy, with highly skilled, experienced endoscopists (performing more than 200 endoscopies per year), and quality assurance controls according to the European guidelines on quality in screening colonoscopy [32]. The standard practice of colonoscopy tattooing to provide accurate localization of lesions for later identification is performed at the endoscopist criteria, following the clinical protocols of the Gastroenterology Department at Hospital Clínic, Barcelona, Spain, performed in compliance with the European guidelines [32]. Thus, colonoscopy tattooing is performed after removal of 2-cm polyps or larger, and on all suspicious lesions at colonoscopy situated outside of the cecum or rectum: namely non-resectable polyps, lesions suspected of submucosal invasive carcinoma, or endoscopically partially resected advanced adenomas (>10 mm, villous, or after a pathology diagnosis of high-grade dysplasia).

**Fig. 1:**
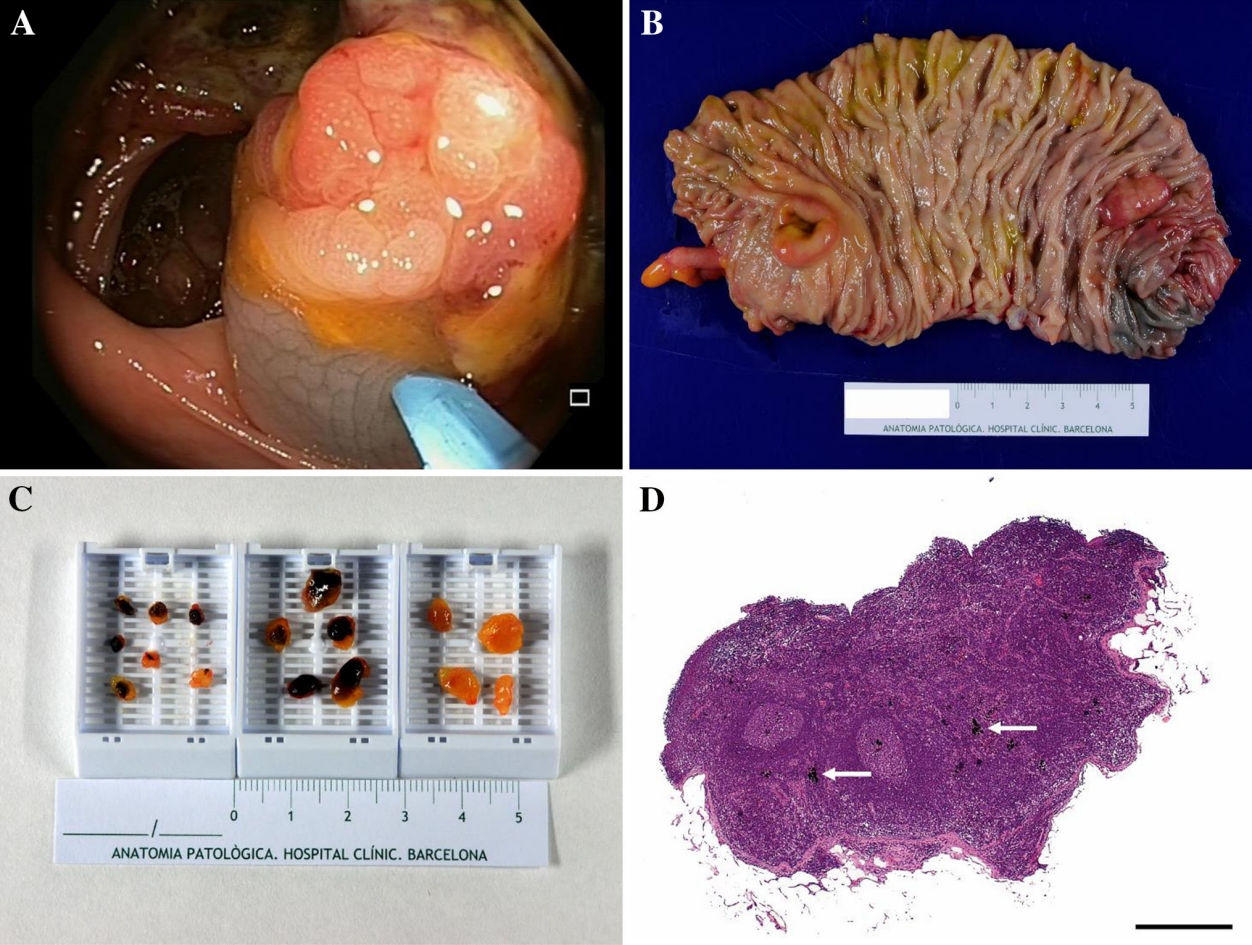
**A** Endoscopic tattooing of a colon carcinoma. Notice submucosal ink injection adjacent to the lesion; **B** gross surgical specimen of a right-side hemicolectomy with tattooed area near an advanced adenoma. Ruler in centimeters; **C** lymph nodes harvested from one surgical specimen. The darker tonalities of tattooed lymph nodes eased their procurement. Notice the left cassette with some small tattooed lymph nodes. A few lymph nodes placed on the right cassette had no ink. Ruler in centimeters; **D** histological HE section of a LN with presence of carbon particles inside (*arrows*) from previous tattooing (*scale bar* 500 µm)

For tattooing, 10 % of diluted and sterilized commercial India ink was used (Pelikan drawing ink color black (A17), Pelikan Vertriebsgesellschaft mbH & Co. KG, Hannover, Germany), which was crafted at the Pharmacy Department of Hospital Clinic by mixing 6 ml of ink and 54 ml of bi-distilled water, divided in 2-mL glass vials, closed, and sterilized by autoclaving at 120 °C for 20 min. India ink tattooing was performed by injection of 1 mL of ink in the submucosa adjacent to the lesion after previous saline submucosal injection, to verify its correct location and to avoid ink injection into the peritoneum (Fig. 1a). For lesions located at the sigmoid or ascending colon, tattoos were placed 1 to 2 cm distally to the lesion and on two opposite sides of the colonic lumen. For lesions located at the transverse colon, tattoos were placed both distally and proximally to the lesion and on both sides of the lumen (i.e., at 3 and 9 o’clock).

#### Surgery

Laparoscopy-assisted colectomy under general anesthesia was performed in all patients by a single gastrointestinal surgical team with wide experience in laparoscopic procedures [4]. Throughout the operation, a pneumoperitoneum with intra-abdominal pressure between 10 and 14 mmHg was maintained. In all patients, colon resection was carried out following oncological criteria, consisting in proximal vessel ligation, en bloc lymphadenectomy, and broad macroscopic resection margins. Routine measures used to prevent port-site metastasis included initial vascular ligation, the use of a wound edge protector, reduction in intra-abdominal pressure before tumor extraction, and exhaustive cleansing with 5 % iodopovidone solution.

#### Surgical specimens

The study included colectomies containing adenocarcinomas, partially resected advanced adenomas, non-resectable polyps, adenomas with suspicion of submucosal invasive carcinoma, and endoscopically resected malignant polyps that contained adverse prognostic factors listed above, which required complete colectomy (Fig. 1B). Pathological data from both endoscopically and surgically resected neoplasms were collected and recorded (Table 1). Patient’s staging resulted from the pathological evaluation of both endoscopic and surgical specimens. Gross processing and pathology reports were performed according to the protocols of the pathology department, adopted from the latest version of the colorectal protocol of the AJCC [33]. All cases were diagnosed and reviewed by two senior gastrointestinal pathologists (JAB, MC).

**Table 1:** Patient demographics and specimen characteristics

Variables	Total	Tattooed specimens	Non-tattooed specimens	*p* value
Cases	71	47	24	
Gender				0.61
Male	43 (60.6)	27 (57.4)	16 (66.7)	
Female	28 (39.4)	20 (42.6)	8 (33.3)	
Age (years)	64 (59–70)	63 (59–68)	66 (62–74)	0.15
Surgical specimen characteristics				
Specimen size (cm)	14 (11–18)	14 (12–17.3)	13.3 (10.9–19.3)	0.93
Adenocarcinoma size (cm)	1.5 (0.9–3)	1.5 (0.9–2.5)	1.8 (0.8–3.4)	0.62
Tumor location				0.12
Cecum	12 (16.9)	4 (8.5)	8 (33.3)	
Ascending colon	16 (22.5)	9 (19.1)	7 (29.2)	
Hepatic flexure	3 (4.2)	3 (6.4)	0 (0.0)	
Transverse colon	6 (8.5)	6 (12.8)	0 (0.0)	
Splenic flexure	5 (7.0)	3 (6.4)	2 (8.3)	
Descending colon	3 (4.2)	3 (6.4)	0 (0.0)	
Sigmoid colon	26 (36.6)	19 (40.4)	7 (29.2)	
Surgical specimen type^a^				0.17
Completely resected	18 (25.3)	15 (31.9)	3 (12.5)	
Partially resected	6 (8.5)	3 (6.4)	3 (12.5)	
Non-resected	47 (66.2)	29 (61.7)	18 (75.0)	
Lymphovascular invasion^b^				0.09
No	65 (91.5)	41 (87.2)	24 (100)	
Yes	6 (8.5)	6 (12.8)	0 (0.0)	
Grade				0.15
High grade	9 (12.7)	8 (17.0)	1 (4.2)	
Low grade	62 (87.3)	39 (83.0)	23 (95.8)	
MS instability	5 (7.0)	2 (4.3)	3 (12.5)	0.33
Tumor budding (*n* = 43)^c^				1.00
High grade	30 (69.8)	21 (70.0)	9 (69.2)	
Low grade	13 (30.2)	9 (30.0)	4 (30.8)	
pTMN				0.53
pT0	8 (11.3)	4 (8.5)	4 (16.7)	
pTis	17 (23.9)	10 (21.3)	7 (29.2)	
pT1	27 (38.0)	20 (42.6)	7 (29.2)	
pT2	19 (26.8)	13 (27.7)	6 (25.0)	

Categorical variables are shown as absolute frequencies and percentages. Numerical variables are described as median and interquartile range (IQR)

^a^With respect to the endoscopic resection

^b^In one case, lymphatic invasion could not be assessed

^c^Tumor budding was assessed in 43 infiltrating carcinomas

#### Lymph node harvest

Lymph nodes from all surgical specimens were identically procured and assessed (Fig. 1C). LNs were also dissected and analyzed from colectomies with no residual tumor performed after an endoscopic polypectomy containing a pT1 carcinoma with adverse prognostic factors. Fresh LN procurement from the mesocolon fat was performed at the pathology department within 50 min after surgical resection by four pathologists, IA, CM, NR, and MC. Fresh LNs were sectioned and submitted for both conventional histology with HE and the OSNA molecular assay, for the detection and amplification of tumor CK19 mRNA [28]. A second-look LN search was performed after 18–30 h of formalin fixation. LNs revealing solutions (i.e., alcohol) were not used. All formalin-fixed paraffin-embedded (FFPE) LNs were submitted only for HE analysis. The presence of India ink in the LN was recorded after evaluation of the HE slides from all LNs using a conventional Olympus BX41 microscope (Olympus, Tokyo, Japan) (Fig. 1D).

#### OSNA lymph node molecular analysis for CK19 mRNA quantification

The OSNA method was performed following the manufacturer’s instructions, using a modified protocol from Tsujimoto et al. [28, 34, 35]. In this assay, the amount of CK19 mRNA/µL copies correlates with the size of the metastasis [34]. The results were based on the number of CK19 mRNA copies/µL obtained for each LN, with a cutoff of 100 CK19 mRNA copies/µL. Values from 100 to 250 CK19 mRNA copies/µL corresponded to isolated tumor cells (ITC). The total tumor load (TTL) of a given specimen resulted from the sum of all CK19 mRNA copies/µL from each positive LN. Evaluation of the molecular results was performed blindly with respect to both clinical and pathological assessments.

#### India ink and OSNA assay interference test

In order to determine whether the carbon particles from India ink interfered with the RT-LAMP reaction, we tested the RT-LAMP CK19 mRNA reaction with and without India ink at 1:100 dilution, which represents a surplus of carbon particles compared to the real traces of carbon particles present in the LN of a tattooed surgical specimen. From a 2000 µL mix containing 20 µL of the positive control containing human CK19 mRNA sample and 1980 µL of lysis buffer Lynorhag (Sysmex Corp. Kobe, Japan), we transferred 200 µL of the mix into 10 OSNA vials and performed the analysis using the Lynoamp BC gene amplification reagent (Sysmex Corp. Kobe, Japan). The same process was done with 20 µL of India ink, 20 µL of positive control CK19 mRNA sample, and 1960 µL of Lynorhag (Sysmex). The amplification product was detected by measuring the rise time required to exceed a predetermined threshold turbidity caused by the by-product magnesium pyrophosphate. The values obtained among samples with high concentration of India ink and without India ink were not significant, showing the absence of India ink interference in the RT-LAMP reaction (Supplementary figure).

### Outcomes

The main outcome was to compare the presence of CK19 mRNA tumor burden in LN among tattooed and non-tattooed specimens.

The secondary outcome was to determine the differences in the yield of LN procured in tattooed and non-tattooed specimens.

### Statistical analysis

Continuous variables were described as median and interquartile ranges (IQR), and categorical variables as absolute frequencies and percentages. Spearman’s rank correlation coefficient was applied to assess correlation between continuous variables. The association of categorical variables was assessed by Fisher’s exact test. The Mann–Whitney–Wilcoxon test was performed to analyze the statistical significance of differences in continuous parameters. Cohen’s kappa was used to assess the degree of agreement. A mixed-effects logistic model with a random patient effect was used to assess the prediction of OSNA outcome in tattooed cases. A *p* value of <0.05 was considered statistically significant. All analyses were performed using R statistical environment (V.3.0.2) [36].

## Results

### Sample characteristics

**Fig. 2:**
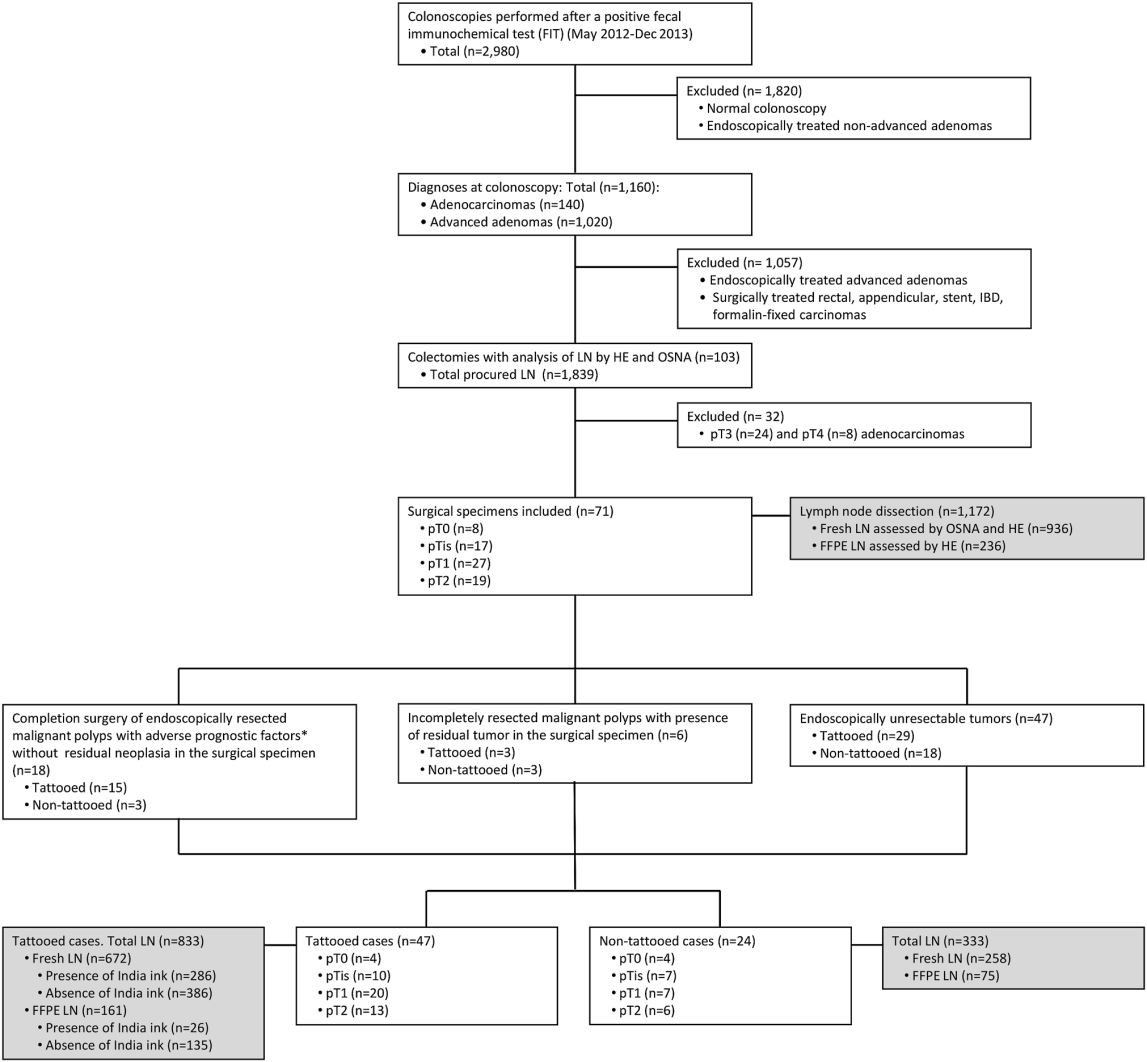
Study flow diagram. Selection and classification of patients according to endoscopic tattooing and pathological findings. *The presence of at least one of the following features: poor differentiation, lymphovascular invasion, high-grade tumor budding, tumor margin ≤1 mm, submucosal invasion >2 mm

The flowchart of the study is detailed in Fig. 2. A total of 2980 colonoscopies were performed on patients with a positive FIT result. We excluded 1820 patients who had a normal colonoscopy or non-advanced adenomas which were endoscopically treated. We found 140 CRC and 1020 advanced adenomas. Most of the latter were endoscopically treated. A total of 103 surgically treated cases were included for LN analysis with OSNA and HE. Of them, 32 pT3–4 carcinomas were excluded. Finally, 71 patients met the study selection criteria. These individuals comprised of (a) 18 patients with endoscopically resected malignant polyps with adverse prognostic factors submitted to surgery. No residual tumor was found in the colectomy specimen; (b) 6 patients with partially resected malignant polyps at colonoscopy, with the presence of residual tumor in the surgical specimen; and c) 47 patients with endoscopically unresectable tumors.

Colectomy specimens were 14 cm (IQR 11–18 cm) in average size. Forty-seven (66.2 %) cases were tattooed and 24 (33.8 %) non-tattooed. The median period of time between endoscopic tattooing and surgery was 63 days (IQR 38–92 days). No differences were observed between both groups regarding demographic and pathologic specimen characteristics (Table 1). The median adenocarcinoma size was 1.5 cm (IQR 0.9–3.0 cm). Two cases had LN metastases on HE analysis. Regarding classical high-risk factors, 9 carcinomas contained high-grade areas and 6 presented angiovascular invasion. Perineural invasion was not observed in any case.

### Lymph node assessment

The number of LN assessed in this study is detailed at the bottom of the flowchart (Fig. 2) and in Table 2. From the 71 surgical specimens included, 1172 LNs were procured; 936 (79.9 %) were freshly dissected and analyzed for both OSNA and HE. After formalin fixation, 236 (20.1 %) LNs were obtained and analyzed with HE. A median of 15 lymph nodes was obtained per patient, 12 of them freshly harvested. The number of total LN procured per case was significantly higher in tattooed cases (median, 17 LNs in tattooed specimens vs. 14.5 LNs in non-tattooed specimens; *p* = 0.019) (Table 2).

**Table 2:** Lymph node characteristics per case

Variables	Total (*n* = 71)	Tattooed specimens (*n* = 47)	Non-tattooed specimens (*n* = 24)	*p* value
Total lymph nodes	15 (12–20)	17 (13–21)	14.5 (10–17)	0.019
Fresh lymph nodes	12 (9–16.5)	13 (10–18)	10.5 (7.7–13.2)	0.02
FFPE lymph nodes	2 (1–5)	2 (1–5)	3 (1.7–4.2)	0.96
Lymph node harvest time (min)^a^	30 (20–38.5)	30 (20–38.5)	27.5 (20–36.2)	0.91
Lymph node harvest time (min) adjusted per LN	2.2 (1.8–3.0)	2.1 (1.8–2.5)	3.2 (1.9–3.8)	0.014
CK19 mRNA detection				0.61
CK19 mRNA detected	42 (59.2)	29 (61.7)	13 (54.2)	
CK19 mRNA not-detected	29 (40.8)	18 (38.3)	11 (45.8)	
TTL^b^	1350 (640–2938)	1420 (700–4270)	1270 (620–2200)	0.76

Categorical variables are shown as absolute frequencies and percentages. Numerical variables are described as median and interquartile range (IQR)

*FFPE* formalin-fixed paraffin-embedded

^a^Time spent on fresh lymph node harvesting per case

^b^Total tumor load (TTL) was calculated as the sum of CK19 mRNA copies/μL from all positive lymph nodes in one given case. The median and IQR shown was obtained from the cohort of positive CK19 mRNA cases (*n* = 42)

Fresh LN procurement was performed within a median of 30 min (IQR 20.0–38.5 min). Although no differences were found in time expended on LN harvesting among tattooed and non-tattooed cases, a significant reduction in the harvesting time was observed in the former, when LN search time was corrected by the number of LN collected (*p* = 0.014, Table 2).

### Analysis of the presence of India ink and tumor CK19 mRNA in LN among tattooed cases

We assessed with the optical microscope the presence of traces of India ink in the form of carbon particles among the 833 LNs obtained from the 47 tattooed cases; 672 LNs were freshly collected. India ink was present in a total of 312 LNs (286; 42.6 % fresh LNs, and 26; 16 % FFPE), See flowchart in Fig. 2. Carbon particles were present in a median of 7 LNs (IQR 4–8) per case.

Twenty-nine tattooed patients were positive for CK19 mRNA (61.7 %). We analyzed the association between the presence of India ink in LN and the detection of tumor CK19 mRNA (Table 2). Of the 672 freshly harvested LNs, 72 (10.7 %) contained tumor CK19 mRNA (44 LNs with India ink and 28 without). Importantly, 15.3 % (44/286) of LNs with carbon particles contained tumor CK19 mRNA, while less than 7.3 % (28/386) of LNs without India ink were positive for CK19 mRNA (Table 3). The logistic model with a random patient effect gave a significant effect of ink (*p* < 0.001) in the CK19 mRNA detection with an odds ratio of 3.1 (95 % CI 1.7–5.5).

**Table 3:** CK19 mRNA detection in tattooed and non-tattooed lymph nodes

	Total LN no. (%)	Tattooed LN no. (%)	Non-tattooed LN no. (%)	
CK19 mRNA detected	72 (100)	44 (61.1)	28 (38.9)	*p* < 0.001
CK19 mRNA not-detected	600 (100)	242 (40.3)	358 (59.7)	
Total LN	672	286	386	

*LN* lymph node

### Analysis of CK19 mRNA in the whole cohort

CK19 mRNA assessment revealed the presence of traces of tumor CK19 mRNA in LN of 42 out of 71 cases (59.2 %). The median TTL was 1350 (IQR 640–2938) CK19 mRNA copies/µL. Two tattooed cases with LN metastases on HE had a significantly higher TTL of 560,000 and 41,160 CK19 mRNA copies/µL, respectively. The median TTL of patients with histologically negative LN was 1275 CK19 mRNA copies/µL (IQR 620–2262). Additional analysis between OSNA results and classical high-risk factors showed an association with tumor size (positive cases showed larger tumors (*p* = 0.02)) and higher tumor grade (*p* < 0.01) (data not shown).

### Treatment and clinical follow-up

All patients were surgically treated by a high-volume practice surgical team specialized in laparoscopic CRC surgery. The two patients with positive LN on HE received adjuvant chemotherapy. Median follow-up was 984 days (IQR 801.5–1118.5 days). Two patients developed distant metastases at 4 and 21 months after surgery, respectively. Both patients presented pT2N0 right-sided tumors with no high-grade features and comparable tumor sizes (30 and 25 mm). The first patient was not tattooed, with 14 out of 18 LNs analyzed by OSNA, and a high TTL of 47,760 CK19 mRNA copies/µL. The second patient was a tattooed case, negative for CK19 mRNA detection, but had only 10 LNs analyzed by OSNA out of 20 procured LNs.

## Discussion

Colonoscopic submucosal tattooing was originally developed to aid in the localization of colon tumors by the endoscopist and surgeon. Although it has been a useful clinically oriented method [1–4], its impact on and benefit in relation to LN retrieval and CRC staging is controversial [9, 20–25]. While some studies find that preoperative tattooing improves LN harvest [9, 20, 21, 24], other publications with divergent results have recently appeared [22]. It is widely accepted that the number of pathologically assessed LN is a critical issue, as it has been demonstrated that it correlates with survival, particularly for node-negative colon cancer patients. In fact, the greater the number of LN examined, the greater the chance to detect metastases [7–9]. Current guidelines recommend that at least 12 LNs should be pathologically assessed to ensure an adequate specimen evaluation and a reliable pathologic staging [33, 37].

Although the number of LN obtained depends on patient, surgical and pathological factors, the latter are decisive and may include revealing solutions that ease LN procuring, i.e., ether and alcohol. [7, 9, 38]. It seems that placement of tattoo by injection of carbon-based dyes causes a permanent deposit of carbon particles in LN, resulting in a dark stain of the LN which helps gross detection and dissection, especially of small-sized LN. Of notice, the median time period between endoscopic tattooing and surgical resection in our study was of 63 days, with a correct microscopic analysis of the presence of carbon particles, being in other series from intraoperative injection to 30 days between both events [20–22, 24].

In agreement with previous studies [9, 20, 21, 23–25], we have evaluated the usefulness of presurgical colonoscopic tattooing to obtain higher LN retrievals. We obtained a median of 17 LNs in tattooed colectomies compared to 14.5 LNs in non-tattooed ones (*p* = 0.02). We obtained at least 12 LNs in 87.3 % of tattooed cases, in compliance with international guidelines [33], which represented an increase of 20.6 % with respect to non-tattooed cases.

Nodal metastases in early CRC are often present in small, difficult to identify, LN of <5 mm in greatest diameter [17, 18, 33]. Presurgical colonoscopic tattooing enabled us to easily detect those LNs. The presence of tattooing may also enhance the likelihood of harvesting the first level of nodal drainage, as well as other LNs in the drainage basin that can also shelter metastatic disease [9, 20, 23, 25]. Spatz et al. [23] analyzed 311 LNs from 21 specimens concluding that colonoscopic tattooing is potentially beneficial for appropriate colon cancer staging.

Our results reinforce previous findings, having found a significantly higher amount of tumor CK19 mRNA among tattooed LNs, with an odds ratio of 3.1 (*p* < 0.001). In our study, we tried to evaluate the practical benefit of a tattooed specimen. As Bartels did, we considered cases to be tattooed only when they had traces of India ink either at gross or at LN microscopic analysis [20]. Differences in methodology and limitations of previous studies may account for controversial results. Feo et al. [22] retrospectively studied 250 colorectal specimens divided into two cohorts, with and without preoperative colonoscopic tattooing. They concluded that preoperative tattooing did not improve the LN yield. Nevertheless, histologic assessment seeking for LN carbon deposits was performed in only 30 out of 107 tattooed cases. They found LN carbon particles in only 2 of the 30 cases [22]. In our prospective cohort, all 47 tattooed cases had gross and histologic traces of carbon particles.

The prognostic significance of LN tumor burden detection using molecular techniques has already been established for breast and CRC [12–16, 26, 39]. Several meta-analyses have found an independent significant association between molecular LN tumor detection and an increased risk of disease recurrence and poor survival in CRC patients [12–15, 19, 40]. Nevertheless, tumor burden detected using molecular techniques may not always be clinically relevant. A study using RT-qPCR detection of guanylyl cyclase C in colon cancer patients found positive LN in 87.5 % of them, although only 20.9 % developed recurrent disease [13]. Other highly sensitive molecular techniques, such as liquid biopsy, have also demonstrated the presence of circulating cell-free tumor DNA in patients with precursor lesions and in situ carcinomas [41, 42].

The aim of our study was not to prove the prognostic significance of LN tumor burden detection using molecular techniques, but to demonstrate the usefulness of tattooing in the detection of those LN most prone to hold tumor burden. For that reason, we included in our analysis those LN holding isolated tumor cells, or small traces of tumor CK19 mRNA, which are known to date to have no clinical significance. In other studies using OSNA, the presence of small amounts of TTL is overlooked. In fact, TTL over 15,000 CK19 mRNA copies/μL has been settled for breast and thyroid cancer sentinel LN studies to determine the likelihood of additional nodal metastases [26, 31]. In our study, the TTL obtained was much lower, with a median of 1350 CK19 mRNA copies/µL per patient. Interestingly, patients with high TTL had either disease recurrence or LN metastases, except for one patient with disease recurrence that had only 50 % of the retrieved LN assessed by OSNA. Forthcoming studies are needed in CRC to determine the amount of clinically significant nodal total tumor load.

Our study has some drawbacks. Firstly, it was performed in a single institution and has a small number of cases analyzed, although it was performed in a screening-based program which allowed us to obtain early-stage carcinomas. Secondly, the distance of the tattoo from the neoplastic lesion was not recorded and, therefore, the correlation between this variable and the CK19 mRNA values could not be ascertained.

Our study highlights colonoscopic tattooing as a highly efficient LN procurement. Tattooing helps the endoscopist and the surgeon to localize the tumor. In addition, it can be used as an extra pathology tool to harvest a higher amount of LN, but more importantly, it makes it possible to find those LNs which might shelter tumor. In a CRC screening-based population, an expanded use of presurgical endoscopic tattooing could benefit patient’s diagnosis and therapeutic management.

## Electronic supplementary material

Below is the link to the electronic supplementary material.

Supplementary material 1 (TIFF 137 kb)
