# Supplementary figures and images for: Endoscopic tattooing of early colon carcinoma enhances detection of lymph nodes most prone to harbor tumor burden
Source: Surg Endosc. 2016 Jun 20;31(2):723–33. doi: 10.1007/s00464-016-5026-3 (PMC5266760; doi:10.1007/s00464-016-5026-3)

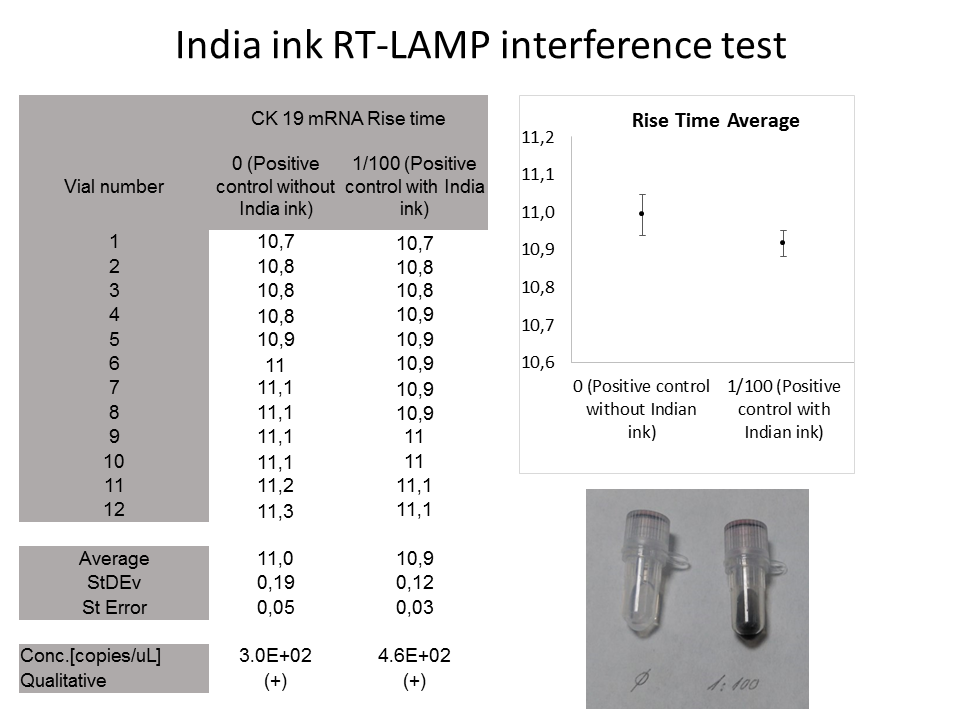

Supplement: Supplementary file 1 — Supplementary material 1 (TIFF 137 kb) [file 464_2016_5026_MOESM1_ESM.tif]
